# A clinically useful risk-score for chronic kidney disease in HIV infection

**DOI:** 10.7448/IAS.17.4.19514

**Published:** 2014-11-02

**Authors:** Amanda Mocroft, Jens Lundgren, Michael Ross, Matthew Law, Peter Reiss, Ole Kirk, Colette Smith, Debbie Wentworth, Jacquie Heuhaus, Christophe Fux, Olivier Moranne, Phillipe Morlat, Margaret Johnson, Lene Ryom, on behalf of the Data on Adverse Events (D:A:D) study group,the Royal Free Hospital Clinic Cohort and the INSIGHT study group

**Affiliations:** 1 Department of Infection and Population Health, University College London, London, UK; 2 Copenhagen HIV Programme, Department of Infectious Diseases, Rigshospitalet, University of Copenhagen, Copenhagen, Denmark; 3 Division of Nephrology, Mount Sinai School of Medicine, New York, USA; 4 The Kirby Institute for Infection and Immunity, University of New South Wales, Sydney, Australia; 5 Division of Infectious Diseases, Department of Global Health, Academic Medical Centre, University of Amsterdam, Amsterdam, Netherlands; 6 Division of Biostatistics, University of Minnesota, Minnesota, USA; 7 Clinic for Infectious Diseases and Hospital Hygiene, Kantonsspital Aarau, Aarau, Switzerland; 8 INSERM U 897, CHU de Bordeaux, Université Bordeaux Segalen, Bordeaux, France; 9 Department of Nephrology, Public Health Department, CHU Nice, Nice, France; 10 Thoracic Medicine, Royal Free Hospital NHS Trust, London, UK

## Abstract

**Introduction:**

Development of a simple, widely applicable risk score for chronic kidney disease (CKD) allows comparisons of risks or benefits of starting potentially nephrotoxic antiretrovirals (ARVs) as part of a treatment regimen.

**Materials and Methods:**

A total of 18,055 HIV-positive persons from the Data on Adverse Drugs (D:A:D) study with >3 estimated glomerular filtration rates (eGFRs) >1/1/2004 were included. Persons with use of tenofovir (TDF), atazanavir (ritonavir boosted (ATV/r) and unboosted (ATV)), lopinavir (LPV/r) and other boosted protease inhibitors (bPIs) before baseline (first eGFR >60 ml/min/1.73 m^2^ after 1/1/2004) were excluded. CKD was defined as confirmed (>3 months apart) eGFR <60. Poisson regression was used to develop a score predicting low (<0 points), medium (1–4 points) and high (>5 points) risk of developing CKD. Increased incidence of CKD associated with starting ARVs was modelled by including ARVs as time-updated variables. The risk score was externally validated on two independent cohorts.

**Results:**

A total of 641 persons developed CKD during 103,278.5 PYFU (incidence 6.2/1000 PYFU, 95% CI 5.7–6.7). Older age, intravenous drug use, HCV+ antibody status, lower baseline eGFR, female gender, lower CD4 nadir, hypertension, diabetes and cardiovascular disease predicted CKD and were included in the risk score (Figure 1). The incidence of CKD in those at low, medium and high risk was 0.8/1000 PYFU (95% CI 0.6–1.0), 5.6 (95% CI 4.5–6.7) and 37.4 (95% CI 34.0–40.7) (Figure 1). The risk score showed good discrimination (Harrell's c statistic 0.92, 95% CI 0.90–0.93). The number needed to harm (NNTH) in patients starting ATV or LPV/r was 1395, 142 or 20, respectively, among those with low, medium or high risk. NNTH were 603, 61 and 9 for those with a low, medium or high risk starting TDF, ATV/r or bPIs. The risk score was externally validated on 2603 persons from the Royal Free Hospital clinic cohort (94 events, incidence 5.1/1000 PYFU; 95% CI 4.1–6.1) and 2013 persons from the control arms of SMART/ESPRIT (32 events, incidence 3.8/1000 PYFU; 95% CI 2.5–5.1). External validation showed consistent CKD rates across risk groups (Figure 2).

**Interpretation:**

Traditional and HIV-related risk factors were predictive of CKD; all are routinely available, making the risk score easy to incorporate into clinical practise and of direct relevance for clinical decision making. NNTH in persons starting potentially nephrotoxic ARVs at high risk of CKD were low, and alternative ARVs may be more appropriate.

**Figure 1:**
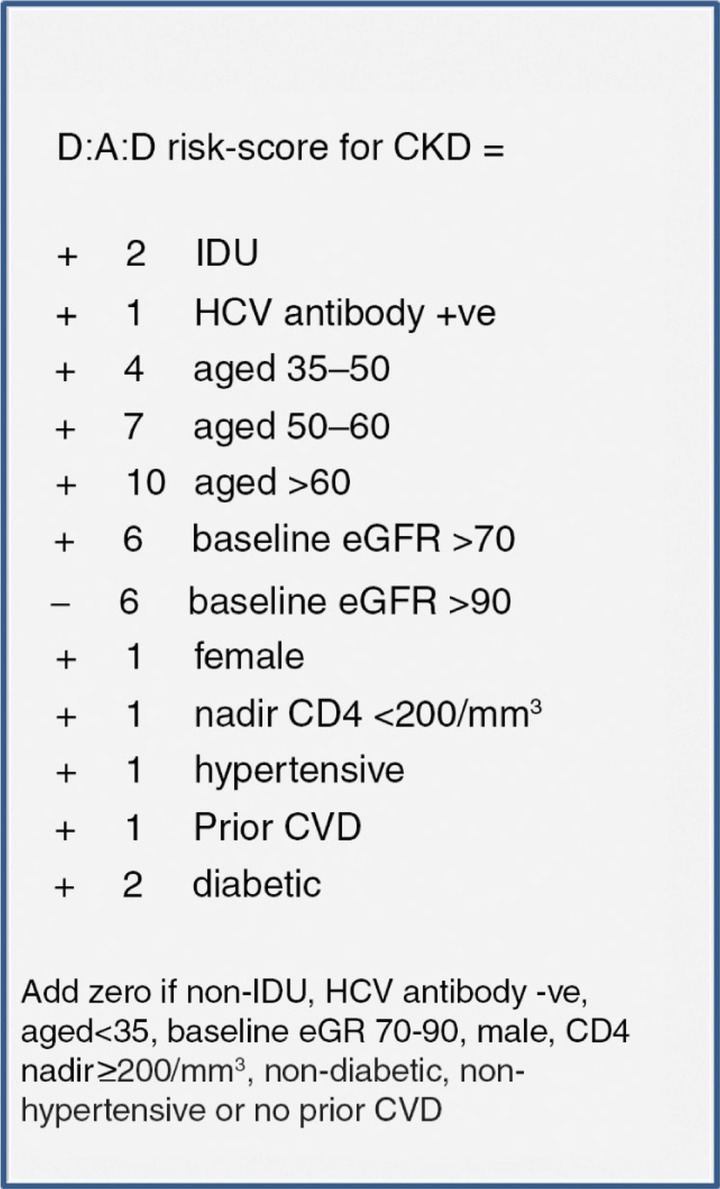
D:A:D risk-score for CKD.

**Figure 2:**
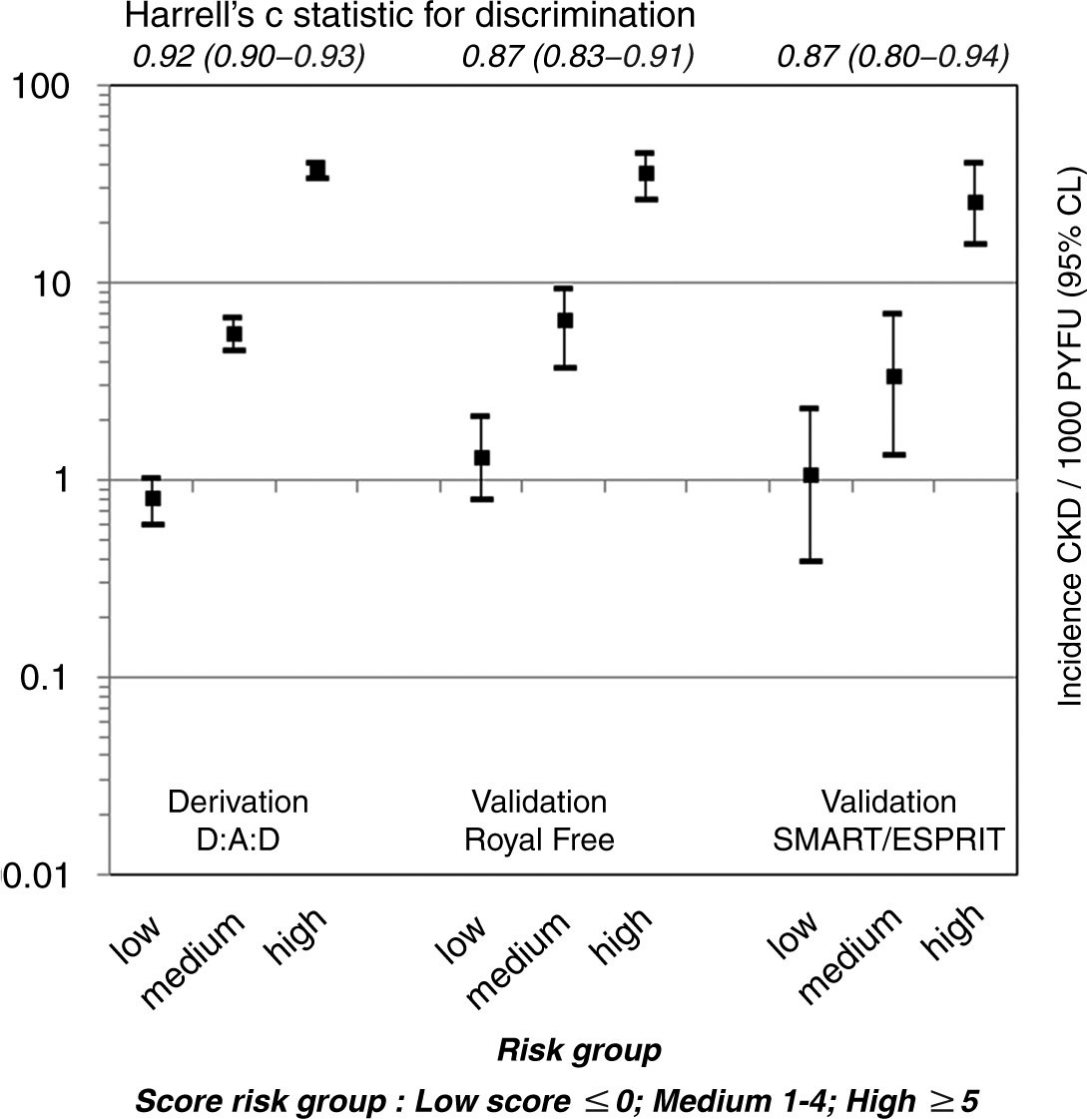
Incidence of CKD in D:A:D, the Royal Free Hospital Cohort and INSIGHT according to low, medium and high risk of CKD.

